# Excellent mid- to long-term survival of tantalum metal cones in a case series of revision knee arthroplasty with severe bony defects

**DOI:** 10.1007/s00167-023-07593-8

**Published:** 2023-10-11

**Authors:** Michael Eder-Halbedl, Andrea Fink, Martin Pietsch, Oliver Djahani, Siegfried Hofmann

**Affiliations:** 1 Department of Orthopedics and Traumatology, LKH Feldbach-Fürstenfeld, Ottokar-Kernstock-Straße 18, 8330 Feldbach, Austria; 2 Department of Orthopedics and Traumatology, Medical University of Graz, Auenbruggerplatz 5, 8036 Graz, Austria; 3 Department of Orthopedics, LKH-Murtal, Stolzalpe, Stolzalpe 38, 8852 Murau, Austria

**Keywords:** Tantalum metal cones, Knee revision arthroplasty, Hybrid cemented, AORI, Bone defects, Knee

## Abstract

**Purpose:**

Severe metaphyseal bone defects remain a challenge and represent a growing problem in revision total knee arthroplasty (RTKA). The purpose of this study was to examine the survival of first-generation tantalum metal cones (TMC) and to assess clinical and radiographic data obtained from mid- to long-term follow-ups (FU) after RTKA with severe bony defects.

**Methods:**

This retrospective case series included 100 consecutive patients of the same centre, who underwent RTKA surgery with TMC for tibia and/or femur bone defects between January 2011 and December 2015. Fourteen patients had died and six were lost for FU, leaving a total of eighty patients (one hundred and twelve TMC) for final evaluation. Clinical parameters including the Knee Society Score (KSS), visual analogue scale (VAS), Western Ontario and McMaster Universities Osteoarthritis Index (WOMAC) and range of motion (ROM) were determined preoperatively based on the patients’ medical charts, and assessed again during the final FU after an average of 6.1 (5–9) years postoperative. Standardised postoperative X-rays were analysed during the final FU visit for osseointegration of the cones, and any signs of implant loosening were assessed with the modified Knee Society Radiographic review criteria. Perioperative and postoperative complications, reoperations, as well as implant and cone re-revisions were analysed. Survivorship analysis was performed with (a) cone-related revision for any reason and (b) implant component revision for any reason.

**Results:**

Previous RTKA had to be performed due to 64 (80%) aseptic and 16 (20%) septic failures. At the final FU, 75 (94%) tibia and 76 (95%) femur TMCs and implants were clinically stable. One patient experienced loosening of cones and implants at the femur and tibia but denied re-revision surgery. There were eight (10%) reoperations including two early wound healing problems, two inlay changes, two periprosthetic fractures, one debridement, antibiotics and implant retention (DAIR), and one secondary patella replacement. The six (7.5%) re-revisions included two aseptic loosening’s of the opposite implant without TMC, one arthrodesis for recurrent instability, and three deep infections managed by two two-stage exchanges, and one amputation for persistent infection. At re-revision, all TMC cones were osteointegrated without signs of loosening. The determined clinical parameters showed significant (*p* < 0.001) postoperative improvement, and objective KSS was rated as excellent in 51%, and as good in 22% of patients at the final FU. The estimated 8-year Kaplan–Meier survival was 95% for TMC and 92.5% for implant components.

**Conclusion:**

Tantalum metal cones (TMC) demonstrate a secure fixation for treatment of severe femoral and tibial metaphyseal bone defects during RTKA. This fixation concept showed excellent mid- to long-term clinical and radiographic outcomes with promising 8-year survival rates for cones and implant components.

**Level of evidence:**

Retrospective cohort study, Level IV.

## Introduction

The frequency of revision total knee arthroplasty (RTKA) is growing, and orthopaedic surgeons are increasingly faced with the challenging problem of managing large bone loss, particularly in patients with a history of multiple surgical procedures of the same knee [29, 33, 34]. Thus, the demand for prosthesis with durable methods of fixation is increasing in revision surgery.

The AORI (Anderson Orthopaedic Research Institute) classification is a useful tool for surgeons to describe and guide femoral and tibial bone loss in RTKA [14]. Based on the size and type of the defect, various treatment modalities are available [5, 24, 25]. Tantalum metal cones (TMC) and sleeves have emerged as promising treatment options for handling major AORI type 2–3 defects, where the metaphyseal segment is damaged or deficient, and fixation of the implant in RTKA is difficult [36]. The mechanical properties of TMC, such as high porosity, bone ingrowth, high co-efficient of friction and stiffness, are similar to that of cancellous bone. The TMC optimises the contact with the host bone and enables a biological fixation [13]. In addition, the metaphyseal fixation of TMC in zone 2 increases the rotational stability and plays a key role for the longevity of implant fixation in case of bone deficiencies in TKA surgery [27]. Many previous studies have reported excellent survival rates and promising clinical and radiological outcomes of TMC and newer cones of different material in the short-term FU, but only few studies have examined the mid- to long-term results thereof.

The primary aim of this study was to evaluate the mid- to long-term survival for TMC and implants in a consecutive series of patients who underwent complex RTKA with severe bony defects. The secondary aim included the evaluation of clinical and radiographic outcomes as well as the analysis of complications, reoperations and revisions. The hypothesis of this study was that TMC will maintain a high survivorship of greater than 90% in the mid- to long-term period and will achieve favourable mid- to long-term clinical and radiological outcomes.

## Materials and methods

In total, 100 consecutive patients underwent RTKA using first-generation TMC (tantalum metal cones, Zimmer, Warsaw, Indiana) for femoral and/or tibial bone defects between January 2011 and December 2015. Of these 100 patients, 14 (14%) had died by the time the current study began for reasons unrelated to the surgical intervention and 6 patients (6%) were lost to follow-up. The final cohort included 80 patients with a mean FU period of 6.1 (5–9) years after surgery. The baseline characteristics of these patients are presented in Table 1.

**Table 1:** Baseline characteristics of the cohort

Demographics	Value
Sex female/male, *n* (%)	44 (55%)/36 (45%)
Body mass index kg/m^2^; mean (SD)	29.7 (18–42)
Age at time of surgery, mean (SD)	65.3 (42–83)
ASA score; mean (SD)	2.17 (1–3)
Knee right/knee left, *n* (%)	36 (45%)/44 (55%)

*ASA* American Society of Anesthesiologists

In all patients, standardised preoperative radiographs were performed to classify the defect according to the AORI classification system and to perform preoperative planning based on full leg weight-bearing radiographs. The requirement for TMC (AORI type 2 and 3 only) was determined based on the preoperatively estimated bone loss and during final intraoperative assessment after implant removal. Reasons for revision, AORI classification and implant information were reviewed retrospectively.

### Surgical technique

In all cases, a standardised failure analysis [18] and a three-step surgical technique [37] were performed by one of the three senior authors (HS, PM or DO). In three cases (4%), tibia tubercle osteotomies were performed; two of which for the approach in stiff knees with patella baja and one for removal of a tibia implant with cementless porous keel fixation. The metaphyseal zone was prepared for the cone using a standardised surgical technique with reamers and broaches to optimise the contact with the host bone. Special care was taken for sclerotic bone at the tibia to prevent any fracture during cone implantation. The preferred implant type for AORI type 2 defects was a semi-constrained condylar knee (CCK) prosthesis (NexGen LCCK Zimmer, Warsaw, Indiana), which allows a less constrained posterior stabilised (PS) insert in balanced knees. For AORI type 3 defects, the preferred implant type was a third-generation rotating hinge knee (RHK) (NexGen RHK Zimmer, Warsaw, Indiana), which allows less rotational stress on the metaphyseal construct. The intramedullary canal was reamed to ensure a press-fit cementless stem fixation into the diaphysis. With the exception of five cases (6%), all prosthesis were fixed with a hybrid cementing technique with cementless stems. The preferred stem length was 100 mm. A short cemented diaphyseal stem (35 mm) was used only when the anatomy did not allow the use of longer stems.

Prior to the hybrid cementing of the implant, the porous tantalum cone was placed in proper position and primary press-fit stability was checked with the “pull out test”. Because of weak distal cortical bone in 18 femurs (23%), an additional prophylactic cerclage wire was used before the placement of the TMC. In severe meta-diaphyseal bone loss, with the exception of one patient, a stacking technique with two TMCs was used as an alternative to a megaprosthesis solution. In this double cone technique, a smaller diaphyseal cone was press-fit implanted into the healthy diaphyseal host bone, and a larger metaphyseal cone was cemented on top together with the implant to reconstruct the metaphyseal anatomy [31]

For all infected cases, the antibiotic (AB) therapy was administered for 6 weeks on the recommendation of the infectious disease specialist. Inpatient mobilisation with partial weight-bearing started the day after surgery and was followed by a standardised inpatient or outpatient rehabilitation programme allowing progressive weight-bearing. All patients had a clinical and radiographic FU visit after 6 weeks and full weight-bearing was allowed. Further clinical checkups were performed annually at the hospital or the referring orthopaedic surgeon.

### Clinical and radiographic assessment

Clinical outcomes were reviewed preoperatively from the institutional medical database and assessed again at the final FU. Clinical outcomes consisted of KSS, ROM and VAS. The KSS was further classified as excellent (objective ≥ of 90, function ≥ 85), good (objective from 77 to 89, function from 73 to 84), fair (objective from 65 to 76, function from 56 to 72) or poor (objective < 65, function < 56) [26]. In addition, the joint-specific Western Ontario and McMaster Universities Osteoarthritis Index (WOMAC) was obtained at the time of FU to assess pain, stiffness and function.

The standardised postoperative anteroposterior X-rays, lateral and full leg film were assessed for osseointegration of the cones and any signs of implant loosening using the modified Knee Society Radiographic review criteria, recently published by Behery et al. [6]. The radiographs were evaluated by EHM and HS on a consensus basis. The AP and lateral view of the femur and tibia were divided into 14 zones and the gap of the radiolucent lines for each of the zones was measured in millimetres and summarised for each component. Femoral constructs were classified as stable (< 8 mm radiolucencies), implant at risk (9–19 mm radiolucencies) or loose (> 20 mm radiolucencies or components migration). Tibial constructs were classified as stable (< 9 mm radiolucencies), implant at risk (10–22 mm radiolucencies) or loose (> 23 mm radiolucencies or components migration). Four patients who were unable to attend the last FU in person received a comprehensive questionnaire, were contacted via telephone, and submitted recent radiographs.

### Complications, reoperations and re-revisions

Perioperative and postoperative complications, reoperations and implant and cone re-revisions were analysed through a medical database search of all included patients. Additionally, patients were asked during the FU visit about any previous or current issues with the implant.

### Statistical analysis

Statistical analysis was carried out using IBM® SPSS® Statistics (Version 27.0.1.0). Descriptive data analysis is presented as median and interquartile range (IQR) for continuous variables and as absolute/relative frequency for categorical variables. The Kolmogorov–Smirnov test was used to assess whether the data (KSS, VAS) were normally distributed. To detect significant differences between preoperative and postoperative data, the Wilcoxon matched-pair signed-rank test and paired *t *tests were performed for parametric and non-parametric distributions, respectively. A *p* value of < 0.05 was considered as statistically significant. The Kaplan–Meier method was employed to calculate cumulative unadjusted component survival with (a) cone-related revision for any reason and (b) implant component revision for any reason.

A post hoc power analysis (G*Power version 3.1.9.4) for the difference in preoperative and postoperative clinical scores was performed and revealed a statistical power of greater than 80% with a *p* value of < 0.05.

This retrospective cohort study was approved by the institutional review board of the Medical University of Graz (EK-nr. 32-196 ex 19/20) and informed consent was obtained from all included patients.

## Results

A total of 112 cones were implanted, and in 6 (7.5%) patients, a double cone stacking technique was performed. The reasons for revision surgery were 64 (80%) aseptic and 16 (20%) septic failures. At the time of revision, 19 (24%) knees showed large bony defects of AORI type 2a, 28 (35%) showed type 2b defects and 33 (41%) showed type 3 defects. Details of the implants and used TMC are summarised in Table 2.

**Table 2:** Reason for revision, defect classification according to the Anderson Orthopedic Research Institute classification, implant information

Reason for revision		No. knees (%)
	Aseptic loosening	36 (45%)
	Instability and malrotation	14 (17%)
	Osteolysis due to poly wear	11 (14%)
	Periprosthetic fractures	2 (3%)
	Osteomyelitis	1 (1%)
	PJI (periprosthetic joint infection)	16 (20%)

AORI classification		No. knees (%)
	Type 2a	19 (24%)
	Type 2b	28 (35%)
	Type 3	33 (41%)

Parameter		No. knees (%)
Level of constrained	Condylar constrained—PS insert	20 (25%)
	Condylar constrained—CC insert	20 (25%)
	RHK third generation	39 (49%)
	Megaprosthesis RHK	1 (1%)
Average polyethylene insert thickness		16.50 mm (range 10-24 mm)
Tibial stem length short^a^/standard^b^/long^c^		1/46/33
Femoral stem length short^a^/standard^b^/long^c^		0^d^/41/38
Hybrid cementing		75 (94%)
Full cemented stems		5 (6%)
Prophylactic femur cerclage		18 (23%)
TT osteotomy		3 (4%)

Type and cone size		No. cones (%)
Tibial cone: total 68	Single	64 (57%)
	Two double cones	4 (4%)
Femoral cone: total 44	Single	36 (32%)
	Four double cones	8 (7%)

*PS* posterior stabilised, *CC* condylar constrained, *RHK* rotating hinge knee, *TT* tibial tuberosity, *PJI* periprosthetic joint infection, *AORI* Anderson Orthopaedic Research Institute

^a^Short 35 mm

^b^Standard 100 mm

^c^Long 140 mm

^d^One femur with cone without stem

### Clinical outcome

All clinical parameters showed significant (*p* = 0.001) improvement from preoperative to the last FU and are summarised in Table 3. The KSS improved significantly from 52 (IQR 22) and 45 (IQR 26) before the operation to 90 (IQR 20.0) and 77 (IQR 30.0) after the operation. The objective KSS was excellent in 40 (51%) and good in 17 patients (22%) at the latest FU. The mean maximum knee flexion increased from 90° (IQR 14.0°) preoperatively to 110° (IQR 34.0°) postoperatively (*p* = 0.001). Twelve patients had a preoperative flexion contracture of > 10°. At the time of last FU, one patient had a residual flex contracture of > 10°.

**Table 3:** Clinical and radiographic outcomes preoperative and at last follow up (KSS, VAS, WOMAC, Flexion contracture) KSS was classified by Miralles-Muñoz FA et al. [26]

Clinical outcome^a^	Preoperative median (IQR)	At last follow-up median (IQR)	*p*-value
KSS objective	52 (± 22)	90 (± 20)	< 0.001^*^
KSS function	45 (± 26)	77.5 (± 30)	< 0.001^*^
VAS	7 (± 1)	2 (± 3)	< 0.001^*^
WOMAC	~	69.7 (± 29.6)	
Knee flexion	90 (± 14)	110 (± 34)	< 0.001^*^

KSS objective	Preoperative No. patients (%)	At last follow-up No. Pat. (%)	
Excellent > 90	1 (1%)	40 (51%)	
Good 77–89	4 (5%)	17 (22%)	
Fair 65–76	14 (18%)	8 (10%)	
Poor < 65	59 (76%)	13 (17%)	

KSS function	Preoperative No. patients (%)	At last follow-up No. Pat. (%)	
Excellent > 85	1 (1%)	23 (30%)	
Good 73–84	5 (6%)	22 (28%)	
Fair 56–72	17 (22%)	15 (19%)	
Poor < 56	55 (71%)	18 (23%)	

Flex contracture	Preoperative No. patients (%)	At last follow-up No. Pat. (%)	
5–10°	10 (13%)	2 (2.5%)	
> 10°	9 (11.5%)	1 (1.5%)	
> 20°	3 (4%)	0	

Radiographic outcome		No. implant (%)	
Tibial implant	Stable (≤ 9)	75 (94%)	
	At risk (10–22)	4 (5%)	
	Loose (≥ 23)	1 (1%)	
Femoral implant	Stable (≤ 8)	76 (95%)	
	At risk (9–19)	2 (2.5%)	
	Loose (≥ 20)	2 (2.5%)	
Tibial cone (*n* = 68)	No radiolucent line	62 (91%)	
	Partial radiolucent line	5 (7.5%)	
	Loose	1 (1.5%)	
Femoral cone (*n* = 44)	No radiolucent line	42 (95%)	
	Partial radiolucent line	1 (2.5%)	
	Loose	1 (2.5%)	

*KSS* American Knee Society Score, *WOMAC* Western Ontario and McMaster Universities Osteoarthritis Index, *IQR* Interquartile range

^a^Clinical outcome 78 patients (arthrodesis and amputation excluded)

### Radiographic outcome

**Fig. 1:**
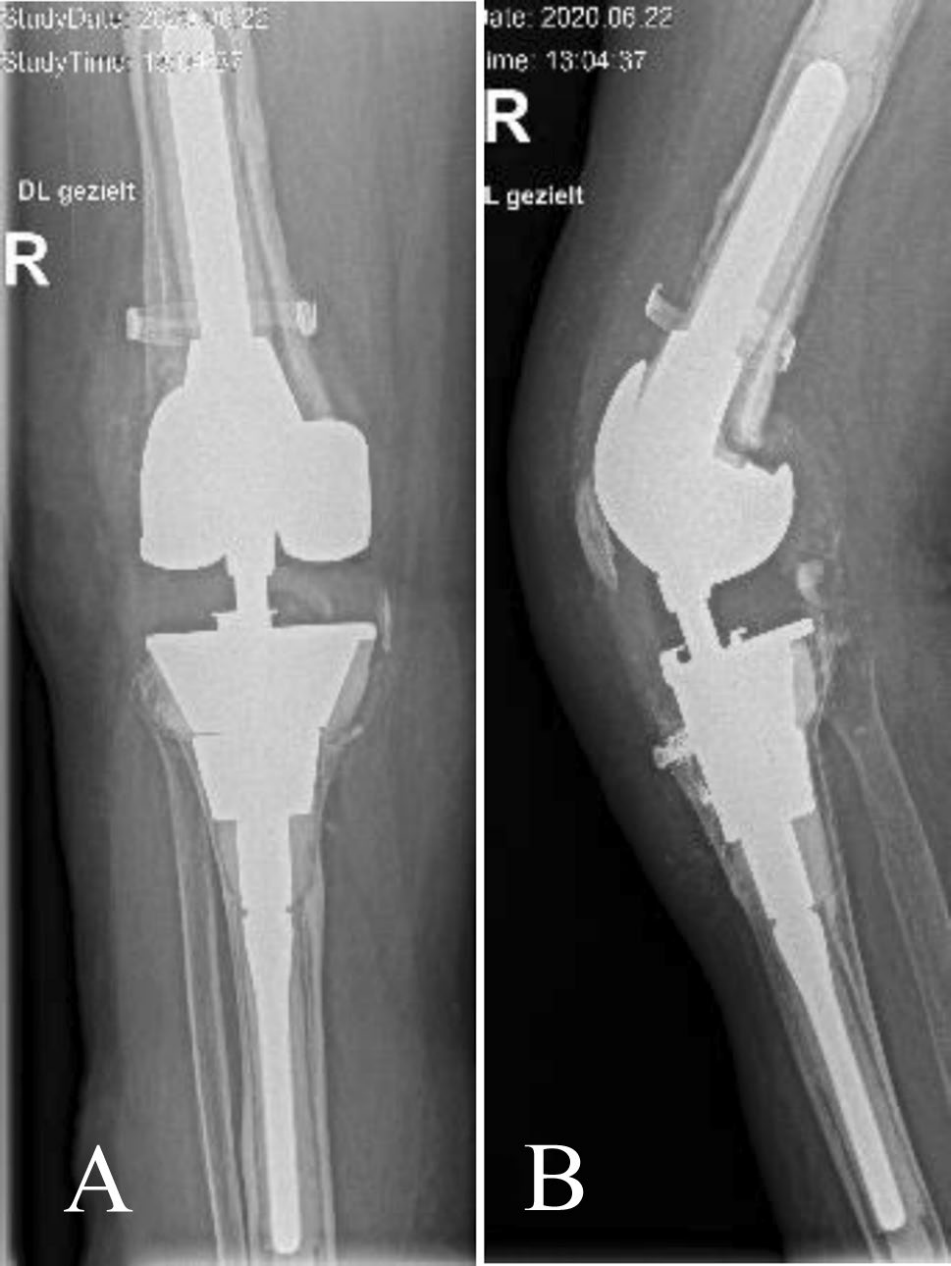
**A** Right knee, anteroposterior, **B** right knee, lateral radiographic views after two septic two-stage revisions at time of fifth revision with tantalum metal cones (TMC). In this knee, both components are loose with double cone technique at tibia, prophylactic cerclage at femur, cemented stems, fixation of the tibial tuberosity osteotomy with two screws into the TMC and complete radiolucent lines at 8.2-year follow-up (FU) (patient who denied re-revision surgery and was calculated as septic failure)

**Fig. 2:**
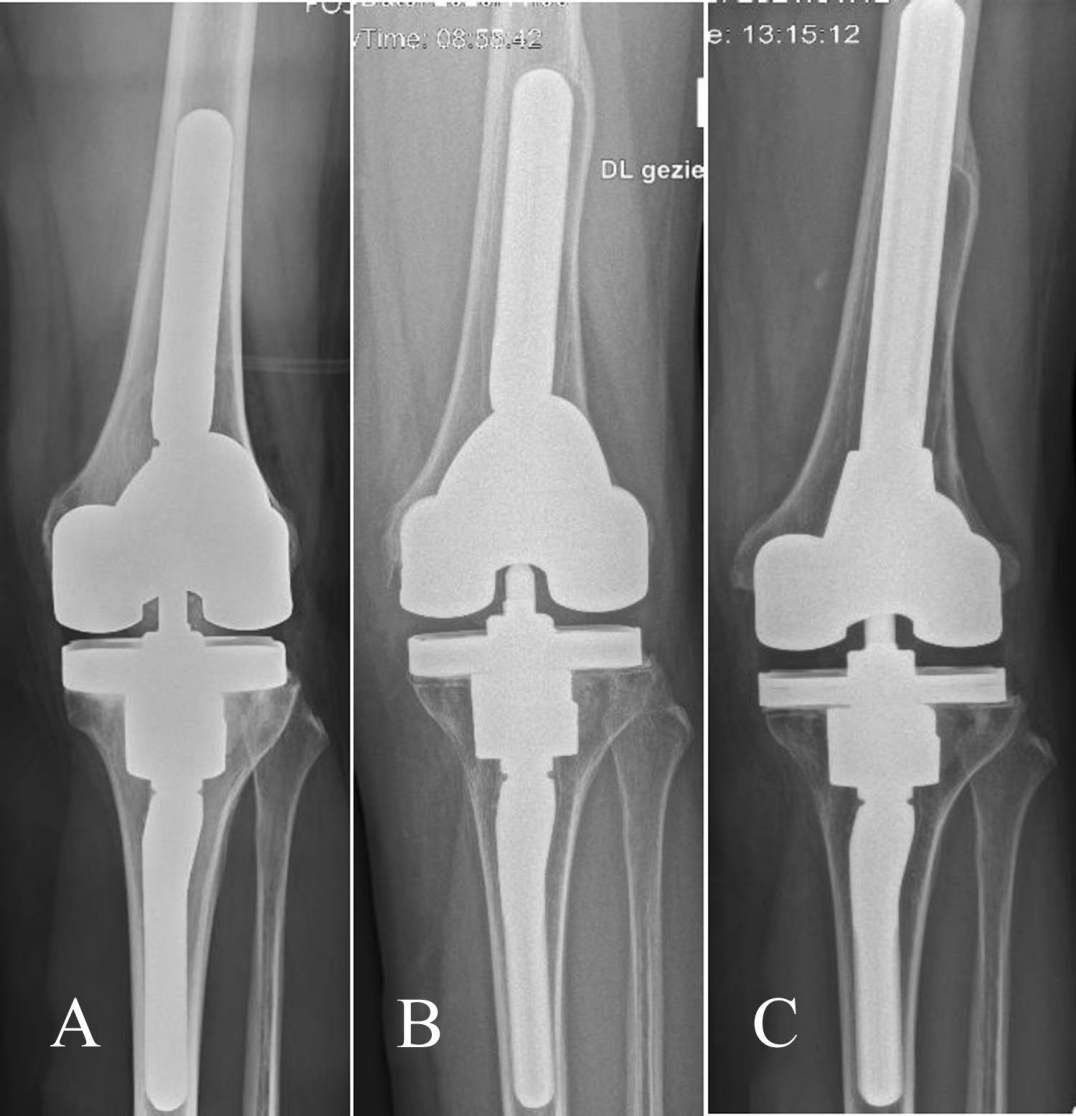
**A** Left knee, anteroposterior radiographs show perfect implantation of a condylar constrained knee (CCK) component with cementless stems and TMC on tibia only. **B** Left knee, anteroposterior radiographs show loose femur component with stable tibia construct for aseptic loosening after 5-year FU. (C) Left knee, anteroposterior radiographs show partial revision of femur component only with TMC using a longer cementless stem

At the final FU, 75 (94%) tibia and 76 (95%) femur TMCs and implants were stable, as summarised in Table 3. Implants at risk were noted in four cases (5%) on the tibial side and in two cases (2.5%) on the femoral side without any clinical symptoms. After the fifth revision surgery with TCM for aseptic loosening following two two-stage septic procedures, one patient displayed femoral and tibial implant and cone loosening with complete radiolucent lines but without any signs of infection. The patient denied revision surgery but was counted as septic failure for the survival calculation (Fig. 1). Two further cases showed a well-fixed TMC but loosening of the opposite implant (one femur and one tibia), which had been initially implanted without a TMC (Fig. 2).

### Complications, reoperations and re-revisions

No intraoperative complications occurred. There were 14 (17.5%) postoperative complications with 8 (10%) reoperations and 6 (7.5%) revision surgeries as summarised in Table 4. The reoperations included two instabilities (one CCK with PS and one RHK) which were treated with isolated inlay exchanges. The PS to CCK insert change was successful, whereas the thicker RHK insert still had recurrent dislocations due to an insufficient extensor mechanism, which eventually resulted in arthrodesis. The two periprosthetic fractures with adequate trauma and stable TMC and implant components were successfully treated with open reduction internal fixation. The two superficial wound healing problems were managed with superficial debridement. One early postoperative deep infection was successfully treated with debridement, antibiotics and implant retention (DAIR). The secondary patella replacement was performed elsewhere with moderate success. The six re-revisions included the two cases described above with aseptic loosening of the opposite implant where no TMC had been used. Both knees underwent successful partial revisions of the loose implant with additional TMC for fixation (Fig. 2). Two of the three late deep infections were treated with two-stage procedures. One knee had to be amputated due to persistent infection after three failed two-stage procedures. Arthrodesis was performed for the previously described RHK case with recurrent dislocations. During re-revision surgery, all four TMCs showed good osteointegration and no signs of loosening.

**Table 4:** Reoperation and re-revision surgery

Reoperations		No. patients (%)
	PS change for CCK inlay	1 (1.25%)
	RHK inlay 1 size thicker	1 (1.25%)
	Sec. patella resurface	1 (1.25%)
	Periprosthetic fracture	2 (2.5%)
	Superficial wound revision	2 (2.5%)
	DAIR	1 (1.25%)

Re-revision surgery		No. patients (%)
Partial revision femur/tibia without cone	Aseptic loosening	2 (2.5%)
Two stage with cone	Deep infection	2 (2.5%)
Arthrodesis with cone	Recurrent instability	1 (1.25%)
Amputation without cone	Recurrent infection	1 (1.25%)

*PS* posterior stabilised, *CCK* condylar constrained knee, *RHK* rotating hinge knee, *DAIR* debridement, antibiotics, and implant retention

### Survivorship

**Fig. 3:**
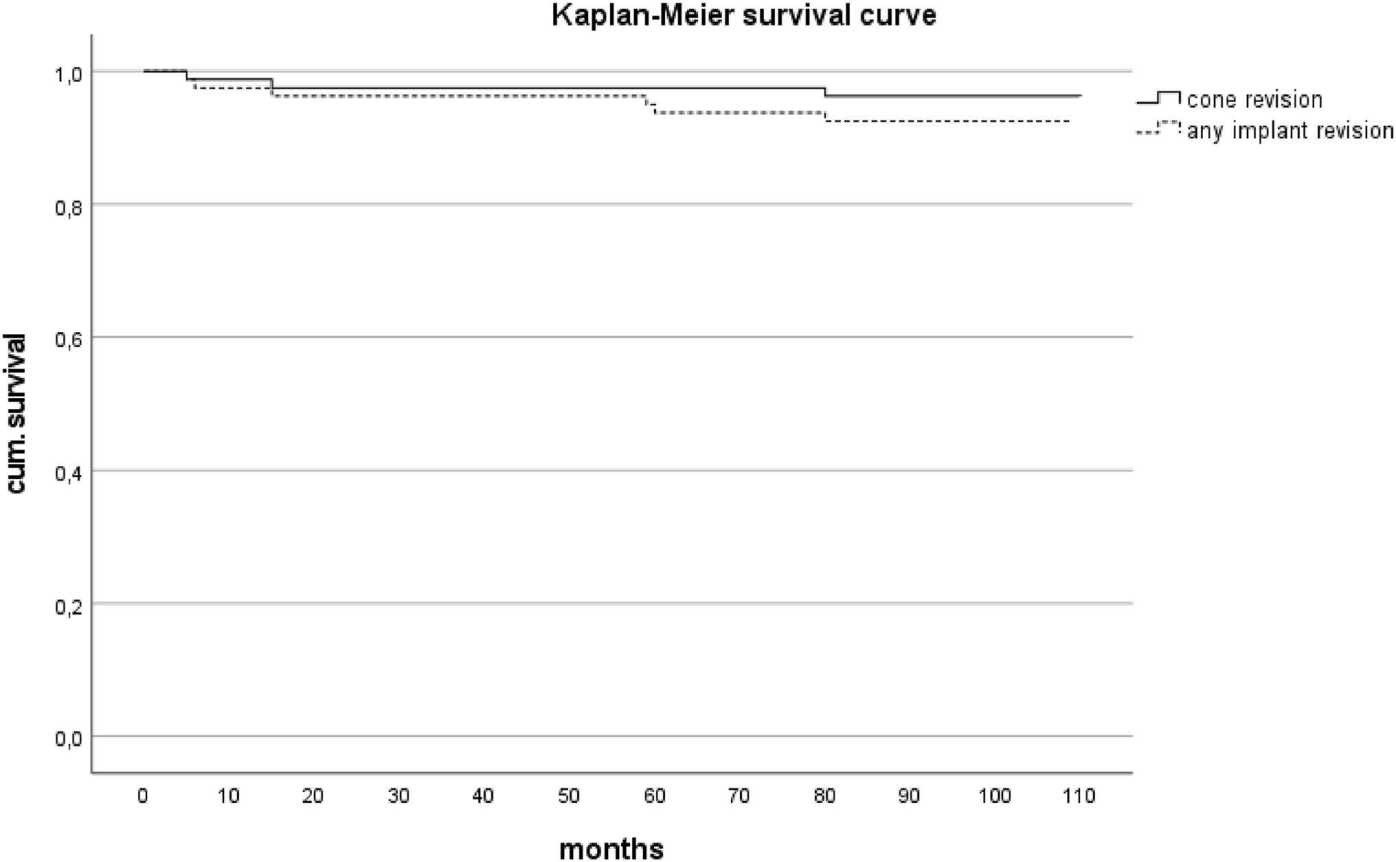
Kaplan–Meier survival analysis for any revision surgery for cones (straight line) and any implant (dotted line)

The Kaplan–Meier estimates demonstrated an 8-year survivorship for TMC revision of 95% for any reason and of 92.5% for implant components (Fig. 3).

## Discussion

The most important finding of the present study is that first-generation TMC in combination with cementless diaphyseal stems demonstrates an excellent mid- to long-term survival rate for both TMC and implant components. In addition, all clinical parameters showed a statistically significant improvement from preoperative to the final FU and are comparable to those of previous studies (Table 5). At the mean FU of 6.1 years, no TMC had to be re-revised due to aseptic loosening and the overall rate of revision and reoperation was low for these complex revision cases.

**Table 5:** Literature review of recent studies on tantalum metal cones

Study	Year	Mean follow-up	Number of patients	Number of cones	Survival cone revision %	Survival component revision %	AORI type 3%	Hinge knee %	Cemented stems %	Radiolucent lines %
Kamath et al	2015	68	63	66	95	94	37	38	94	23
Potter et al	2016	60	157	159	84	–	20	36	100	–
Panda et al	2019	79	79	79	–	95	59	33	–	9
Abdelaziz et al	2019	127	25	32	75	52	36	100	100	–
Abdelaziz et al	2020	50	72	101	83	79	76	100	100	25
Hernandez et al	2021	91	59	62	84	62	24	8	100	30
Rossi et al	2022	90	101	134	–	90	24	–	24	14
De Martino et al	2022	120	30	30	81	75	46	53	10	6
**Present study**	**2023**	**73**	**80**	**112**	**95**	**93**	**41**	**50**	**6**	**5**

*AORI* Anderson Orthopaedic Research Institute

The excellent survival (revision for any reason) of TMC and of implant components was in accordance with four studies documented in the recent literature [12, 20, 28, 32]. In contrast, a few publications report worse mid- to long-term survival rates. For example, Abdelaziz et al. [1, 2] describe two RTKA TMC series. The first report includes infected TKA only, compared to the 20% septic patients in this study cohort, and achieved a lower TMC and implant component survival of 83% and 79% after 4-year FUs, respectively. In the second report, fixed hinges were used in 52% of the patients and a lower cone and implant component survival of 75% and 52% were reported, respectively. The higher failure rates of infected TKA and fixed hinges are mentioned by the authors in both publications. Furthermore, higher aseptic loosening rates of this specific hinge design with large femur diameters are reported in the literature [22].

Potter et al. reported higher failure rates for single femoral cones with AORI type 3 defects compared to the tibia which led to a lower survival rate for femoral cones. Therefore, these authors recommended TMC with different shapes, sizes and methods of preparation for type 3 femur bony defects [30]. Hernandez et al. found a survival rate at a mean FU of 7.5 years for cone and implant components of 84% and 62%, respectively [17]. The lower TMC and implant survival rate in this series was observed due to the higher number (37%) of periprosthetic joint infections (PJI) of patients after two-stage revisions.

Due to the high proportion of type 3 defects (41%) in the present study, half of the cases were treated with RHK. This design allows axial rotation on the tibial plateau, and thus reduces the stress to the interface between the bone and the implant [3]. Moreover, six AORI type 3 defects were successfully treated with a double cone stacking technique in our study and only one megaprosthesis was necessary. The 5% TMC revisions for any reason reported in our study is excellent and finds itself in the lowest range described in the extant literature (Table 5). The patient with loose TMC and components at both tibia and femur (Fig. 1) who denied revision surgery, had a history of two two-stage revisions for infection. Although no infection according to International Consensus Meeting (ICM) guidelines was detected, we suspect this case to be a septic loosening of the TMC and implant components. Two of the three late deep infections were treated successfully with a two-stage procedure. The remaining knee with recurrent infection ended up with amputation after two unsuccessful two-stage procedures.

One of the reasons for the very low infection rate for aseptic revisions in this study might be the AB management of the present study’s institution. Therapeutic AB bone cement with 1 g gentamicin and 1 g clindamycin (Revision bone cement, Biomet, Warsaw, Indiana) is used in combination with prolonged systemic AB therapy for 5 to 7 days for all aseptic revisions. Although this approach with extended AB therapy in these high-risk patients is still controversial and not recommended by ICM 2018 [4], it is currently under debate [8].

It might have been possible to prevent the two (2.5%) cases with partial revision for aseptic loosening of the component without TMC and stable opposite components with TMC (Fig. 2) using a TMC during the primary revision surgery for both components. Ten year ago, the indication for TMC was very restrictive due to the high costs and missing evidence for their benefit, but over time, the number of RTKA has increased from 5 to 15% in the institution the study was conducted in. This trend has been also observed in the United States [10]. Currently, the benefit of TMC, especially for smaller defects, still has to be proven, particularly in comparison to other alternative fixation methods [9, 26, 35].

The long-term fate of the 6 (5%) out of 112 TMC patients with partial radiolucent lines remains unclear. Since none of the patients included in this study showed clinical signs of loosening, they might have remained stable and the long-term outcome will not be compromised. In the authors’ clinical experience, 30% to 50% of the TMCs must be osteointegrated to prevent loosening, but this will have to be proven in biomechanical and clinical studies with more patients and long-term FUs.

In the present study, all cases except for five (6%) were fixed with cementless diaphyseal stems with a hybrid technique. Cemented stems were used only in recurrent infections where the local AB cement in the diaphysis might be beneficial, where the metaphyseal construct seemed not to be stable enough for cementless stem fixation or where short metaphyseal stems were necessary due to meta-diaphyseal anatomic conflicts. This study confirms that diaphyseal cementless fixation with TMC in severe bony defects work very well. Nevertheless, in the published TMC literature (Table 5), cemented stem fixations were used in only 75% of cases. Currently, there is no evidence that one of these fixation philosophies yields better results for RTKA, but many surgeons move to shorter cemented stems together with TMC [19]. It is not clear yet if this approach to reduce stem tip pain works well with AORI type 3 defects [38].

Metaphyseal sleeves constitute a different approach of fixation concept in RTKA with severe bony defects. Sleeves are firmly attached to the prosthesis like a monobloc construct, which might facilitate the use of the implantation technique. However, the reconstruction of the joint line is more limited in sleeves compared to cones. TMCs are an independent part of the prosthesis, and thus are flexible for eccentric bone defects. However, several papers have also shown excellent mid- to long-term survival rates for sleeves [7, 11, 16, 21]. In two recent meta-analyses and one large consecutive series comparing cones and sleeves, the survival rates were comparable [15, 23, 36].

There are some limitations of the present study. First, the data review was performed retrospectively for preoperative clinical outcomes. However, a standardised chart form was used to guide prospective data collection in the local database which enabled a systematic data acquisition. Furthermore, losses to FU or death may introduce an attrition bias, but eventually, a FU of 80% was maintained, which is acceptable for this kind of cohort. Another limitation is the considerably heterogeneous nature of the cohort in terms of indications, numbers of previous surgeries and soft tissue situations. However, as with all re-revisions, each case is somewhat unique. In our study, failure analysis, planning, surgical technique, use of implant constraint and philosophy of implant fixation were standardised and performed by three specialised orthopaedic knee surgeons only, which limits the possible impact of a technical bias. Additionally, there were only limited numbers of patients available for analysis after 8 years, which may bias the estimated TMC and implant survival. For the radiographic review, no inter- and intra-observer analysis has been performed. The major strength of this study is the relatively high number of patients and mid- to long-term FU which allows for valid conclusions regarding the fixation technique used with TMC in combination with cementless stem for severe bony defects.

The excellent mid- to long-term study results of this large series of RTKA using TMC strengthen the promising outcomes of recent literature. Such data are particularly encouraging for patients with a history of multiple previous knee surgeries who may benefit from extended prosthesis survival and improved mobility, as well as reduced pain and stiffness. Moreover, surgeons should attempt to maximise the metaphyseal fixation in AORI type 2–3 defects to guarantee solid primary fixation and a long-term implant survivorship.

## Conclusion

First-generation TMC in combination with cementless stems demonstrate a secure fixation for treatment of severe femoral and tibial metaphyseal bone defects during revision TKA surgery. This fixation concept shows excellent mid- to long-term 8-year survival rates for cones and implant components with favourable clinical and radiographic outcomes.

## Data Availability

Raw data for dataset are not publicly available to preserve individuals’ privacy under the European General Data Protection Regulation.

## Abbreviations

AB: Antibiotic
AORI: Anderson Orthopaedic Research Institute
CCK: Condylar constrained knee
DAIR: Debridement, antibiotics and implant retention
FU: Follow-up
IQR: Interquartile range
KSS: Knee Society Score
LCCK: Condylar constrained knee prosthesis
PE: Polyethylene
PJI: Periprosthetic joint infection
PS: Posterior stabilised
RHK: Rotating hinge knee
ROM: Range of motion
RTKA: Revision total knee arthroplasty
TMC: Tantalum metal cones
TT: Tibial tuberosity
VAS: Visual analogue scale
WOMAC: Western Ontario and McMaster Universities Osteoarthritis Index
ICM: International Consensus Meeting
